# Cutting It Too Fine? The Factor Structure of Fine Motor Skills From Ages 5 to 10 Years

**DOI:** 10.1111/cdev.70016

**Published:** 2025-07-29

**Authors:** Sebastian P. Suggate, Viktoria Karle, Heidrun Stoeger

**Affiliations:** ^1^ Department of Educational Science University of Regensburg Regensburg Germany

**Keywords:** dexterity, factor structure, fine motor skills, graphomotor skills, writing

## Abstract

Fine motor skills (FMS) have been intensely studied in developmental contexts, with little attention to their empirical structure and developmental changes. We tested the factor structure of FMS on 5‐ to 10 year old children in two cohorts from 2020 to 2023, beginning in kindergarten and grade 2 and followed up 1 year later (*n* = 240 and 310, 49.7% female, 74.2% German). Measures assessed dexterity, graphomotor skills, and speed FMS. Exploratory factor analyses were conducted on randomly split‐half samples in kindergarten and grade 2, followed by confirmatory factor analyses on the second of the split‐half samples and the entire cohorts in grades 1 and 3. Findings generally suggest a three‐factor solution (CFIs > 0.95), with indications of gender effects and developmental changes in structure.

A large amount of research evidence has now accrued showing that children's fine motor skills (FMS) relate to cognitive/academic skills (Grissmer et al. 2010; Martzog et al. 2019; Pagani et al. 2010) and developmental delay (Brookman et al. 2013; Lange‐Küttner and Kochhar 2020; LeBarton and Iverson 2013). However, there is often only limited agreement around the definition of FMS, its measurement, and corresponding factor structure, which makes it difficult to (a) interpret previous findings, (b) be confident about current measures, and (c) develop theory on the contribution of FMS to human development and cognition. Few studies adequately address the factor structure, with previous research containing adult samples published over 50 years ago using less refined statistical methods (Fleishman and Hempel 1954; Seashore et al. 1940), small samples (cf., Martzog et al. 2019; Okuda et al. 2019), self‐report measures (Kim et al. 2015; Lin et al. 2014), or an insufficient number of FMS tasks to investigate the structure (Oberer et al. 2017; Schulz et al. 2011; Zoia et al. 2019). Moreover, research has not tested whether the FMS factor structure changes with development, particularly in child samples where schooling takes effect. Accordingly, we examine the factor structure of FMS in children attending kindergarten and primary school (i.e., children aged 5 to 10), testing embodied cognition, functionalism, and developmental changes.

## 1 FMS: Definition and Categorization

Before considering empirical evidence on conceptual structures and underlying constructs, it is useful to consider definitions of FMS, their measurement, and over‐arching theoretical considerations. Thereby, the focus will be on early childhood to adolescence, firstly, because most FMS research has been conducted on this age group and, secondly, because of the developmental and educational role played by FMS (Cameron et al. 2012; Grissmer et al. 2010; Martzog et al. 2019; Suggate et al. 2023).

FMS are generally defined as the ability to perform small and controlled movements with the fingers and hands (Luo et al. 2007; Suggate et al. 2019). FMS, along with gross motor skills, belong to a broader group of (general) motor skills (Hands et al. 2018) and entail a range of processes and subskills, such as integration and coordination with other senses (e.g., vision, proprioception, vestibular, and tactile information), plus underlying strength, fitness, flexibility, and information processing aspects (Chien et al. 2009; Hands et al. 2018; Pont et al. 2009; Suggate 2023). Reflecting the definitional breadth across the literature, a raft of terms has been used as synonyms for FMS, such as: dexterity, visual motor skills, graphomotor skills, psychomotor skill, hand‐eye coordination, manual skills, and to some extent also: tapping, finger agility, finger gnosia, and finger speed.

Turning to tasks used to measure FMS, these, ranked according to approximate level of popularity, include pegboard, maze tracing, tapping, copying, threading and weaving, sorting and building/stacking, and object construction tasks (Carlson et al. 2013; Fleishman and Hempel 1954). Most tasks involve both a speed and accuracy component, for instance copying as many forms as possible correctly within a minute, with some researchers arguing that repetitive, speed‐driven tasks are qualitatively different from tasks requiring more spatially sophisticated movements (Martzog et al. 2019), which in turn differ from those with a direct link to academic skills because they lie close to writing (Suggate et al. 2018). Therefore, we tentatively define three subdomains of FMS more closely, as these appear in much of the literature and can be seen as subskills of FMS based on coherent theoretical approaches outlined below. Dexterity is performance at skillfully manipulating small objects (Backman et al. 1992). Graphomotor skill refers to integrating input from visual and motor modalities to create forms and symbols using writing tools (Bo et al. 2006; Pitchford et al. 2016) to perform small‐scale and precise movements, such as drawing and writing activities (Berninger et al. 1992). Finally, speed‐dominated FMS involve rapid but simple, often repetitive, movements of the fingers and/or hands (Fleishman 1972), such as key‐tapping tasks.

Because FMS are used to perform a nearly infinite range of tasks (e.g., tying shoelaces, gesturing, tool use), it is possible to identify and categorize countless further aspects of FMS. For instance, beginning with the broader concept of hand skills, which is conceptually close to dexterity, Chien et al. (2009) identified 22 different subdomains (e.g., grasping, gesture, holding, calibration, pace, transferring). Focusing on one aspect of manual dexterity, namely in‐hand manipulation of objects, for this subdomain alone, Pont et al. (2009) identified six additional subdomains (e.g., stabilization finger‐to‐palm translation, simple shift, complex shift, simple rotation, complex rotation). Such a detailed conceptualization of FMS has undoubted value, for instance, in occupational therapy settings, but is also conceptually very complex (Hands et al. 2018), perhaps first requiring a series of simpler studies with a broader conceptualization of FMS as a starting point.

## 2 Theoretical Considerations and Implications for the Factor Structure

FMS represent a potentially infinitely complex array of skills, micro and macro movements, with varying degrees of control and purpose (Adolph and Hoch 2019), subjected to different environmental conditions (Thelen 1987, 2000), and hence could likely be categorized according to vastly different criteria (see Chien et al. 2009). Perhaps the first consideration in determining FMS' factor structure is determining the categorization's theoretical framework. One approach that fits with the aims of the current study is to consider FMS in the context of human development, learning, and cognition (Ye et al. 2023). To this end, two main theories seem appropriate as they focus on the role of FMS in children's learning and cognition, namely (a) embodied cognition and (b) functionalism (Suggate and Stoeger 2017), which combined give rise to a third, (c) developmental approach.

### 2.1 Shared‐Processing or Embodied Cognition Theories

Beginning with embodied cognition—or shared‐processing—theories, motor skills are viewed as being intrinsically linked with both the environment and underlying neurological and cognitive skills (Adolph and Hoch 2019; Suggate and Stoeger 2017; Wellsby and Pexman 2014). Such links extend well beyond Piaget's sensorimotor phase in the first 2 years of life (Di Paolo et al. 2014). This proposal has two key tenets. First, cognition has its roots in sensorimotor processes (Kiefer and Pulvermüller 2012; Lakoff and Johnson 2010; Shapiro 2011), of which FMS is a key part (Suggate and Martzog 2021). Second, motor skill is a proxy for the maturity of corresponding sensorimotor areas and processing (Suggate and Stoeger 2017).

Beginning with tenet one, cognition having its roots in sensorimotor processes, it appears relevant that higher‐order skills, such as reading and language, have arisen in a fairly short evolutionary time frame, such that these are unlikely to have their very own processing areas in the brain. Instead, it is believed that reading and language utilize neural areas that were previously devoted to sensorimotor processing (Aboitiz and García 1997; Dehaene and Cohen 2007). Accordingly, it has been proposed that motor areas can be recycled, utilized, or redeployed for use in cognitive processing (Anderson 2007; Dehaene and Cohen 2007; Penner‐Wilger and Anderson 2013; Roebers 2021). This sharing of processing between fine and gross motor and cognitive areas has received strong empirical support in the domain of language (e.g., Kiefer and Pulvermüller 2012; Pulvermüller 2005), mathematics (Link et al. 2013), and cognition (Oberer et al. 2017; Roebers 2021; Roebers et al. 2014), in particular.

The second tenet is that specifically fine motor skills link to cognitive skills, such that better FMS gives rise to better cognition and learning (Penner‐Wilger and Anderson 2013; Suggate and Stoeger 2014, 2017). The idea is that greater FMS represents better formed and more effective corresponding sensorimotor areas, which can then more efficiently support cognitive processing. Support for this comes from numerous studies that show positive links between FMS and mathematics (Fischer et al. 2020; Luo et al. 2007), reading (Cameron et al. 2012; Suggate et al. 2019), language (Brookman et al. 2013; Gonzalez et al. 2019; Suggate and Stoeger 2014), and cognition (Davis et al. 2011; Martzog et al. 2019).

As it applies to the structure of FMS, it would follow that FMS exists as a more global and unitary construct because sensorimotor processes are seen as being amenable to redeployment and recycling (Dehaene and Cohen 2007; Penner‐Wilger and Anderson 2013). For instance, the general ability to manipulate objects and tools with the hand represents the redeployment of similar underlying FMS neural structures and circuits, giving rise to a unitary FMS construct, as the various subskills become integrated or assimilated into larger, more accommodating motor programs (Di Paolo et al. 2014). Finally, it is also interesting to note that, although not framed in terms of embodied cognition or shared motor processes, some motor skill researchers have argued for a general “*g*” factor in motor skills (i.e., gross and fine motor skills; Hands et al. 2018), which would therefore logically apply to FMS as well as part of this general construct.

### 2.2 Functionalism

Functionalist theories point to the role that FMS play in adding to the organism's ability to interact with and, hence, learn from the environment (Iverson 2010; Suggate and Stoeger 2017). Although specific FMS might be useful for certain skills (e.g., thumb index finger closure for turning the page of a book), advantages for cognitive development and learning are most evident when a person can engage in complex environmental interactions, such as those involving objects, tools, and devices, with each of these, in turn, requiring specialized motor programs and skills (Schaal and Schweighofer 2005). Therefore, functionalist theories would conceptualize FMS as having a greater degree of specification than embodied theories do.

The next key question is, what FMS subdomains might exist according to functionalism? One way to approach this division is to consider the kinds of functions that FMS perform in human development. One intuitive category of activities might entail general environmental interactions (e.g., crafting, stacking objects, dicing vegetables, and operating a touch screen). Given the dominance of school, a second apparent category would appear to arise out of academic skills after the onset of school, namely graphomotor skills, which are required for writing. A third category might include speed‐related skills, for instance, partly due to individual differences in muscle performance and fatigue (Forman et al. 2022) and partly due to different environmental experiences (e.g., gaming, touch typing).

### 2.3 Developmental Effects

Finally, it is possible that shared‐processing theories and functionalism both operate and interact developmentally; as children's environments change, so might the factor structure. For instance, children's experiences in preschool and kindergarten are likely more focused on object manipulation and environmental exploration initially but move to graphomotor skills (e.g., Marr et al. 2003; Suggate et al. 2016) and even speed‐related gaming later (Moon et al. 2019). Thus, a functionalist view might see FMS differentiate across development as increasing specialization takes effect. In contrast, a shared‐processing view might argue that skills converge over time as a greater repertoire in FMS develops; greater redeployment of processing across specific networks and motor programs might occur.

## 3 Previous Research on the Factor Structure of FMS

To recapitulate, shared‐processing or embodied theories point toward FMS being a more global construct, with specific subsets depending on the context. Functionalist theories generally predict more specialization, perhaps into dexterity, graphomotor, and speed‐FMS in children. Developmental approaches argue for an interaction between shared‐processing and functionalism, with two possible variants. If a greater functional specialization occurs as children learn more diverse FMS, then the trend might be from a few factors to more across development—particularly after the onset of formal schooling with its focus on graphomotor skills. Conversely, children's FMS might become more unitary as diverse FMS develop redeployable neural structures that can be used for a number of different fine motor tasks, as suggested by embodied theories. We now consider evidence for specific factor models in light of the theories.

Factor analytical research has used both behavioral and self–/parent–/teacher‐report scales. Ideally, data would reflect the performance of children directly, given potential bias in parental or teacher questionnaires (Estevan et al. 2018; Miller et al. 2017; Wilson et al. 2000); however, questionnaires also have the advantage of screening a range of behaviors in a time‐economical fashion. A teacher questionnaire about children's motor skills was administered to the teachers of 144 five‐to seven year old children in kindergarten and first grade (Kim et al. 2015). The 19‐item scale best supported a three‐factor solution, capturing children's classroom FMS, general body awareness (e.g., staying seated in a chair), and difficulty with letters and shapes. Interestingly, the classroom FMS and the letters and shapes scales are both dominated by items that would be classified as graphomotor skills according to our definition, and the body awareness questions contain items that appear to reflect classroom behavior and attention (e.g., takes a long time to find/put things away).

Evidence for five factors was found using a specifically FMS 27‐item questionnaire completed by primary caregivers of 419 grade 1–5 children aged seven to 13 (Lin et al. 2014). One of the factors (pen control) appears to reflect graphomotor skills, and the remaining were aspects of manual dexterity (handcrafts, dining utensils, connecting and separating, opening containers). Thus, these questionnaire studies broadly support a division between graphomotor skills on the one hand and dexterity measures on the other hand, but represent a degree of specificity consistent with functionalism. However, it is also possible that these self‐report estimates reflect a conceptual differentiation on the part of the adults completing the questionnaires rather than a measurable behavioral difference.

Turning to behavioral measures, the Movement‐ABC is a widely used screening measure of motor skills that includes, alongside gross motor and balance, a manual dexterity scale containing a graphomotor task and two manual dexterity measures. Several subsequent factor analyses conducted on kindergarten (Oberer et al. 2017; Schulz et al. 2011; Zoia et al. 2019) and elementary school children (Michel and Gado 2022; Schulz et al. 2011; Zoia et al. 2019) supported the existence of a three‐factor solution that includes two non‐FMS factors (namely gross motor and balance skills), with all FMS measures loading on one factor (cf. Okuda et al. 2019). Although initially appearing to support a single FMS factor as predicted by shared processing, there may have been simply too few FMS tasks (i.e., three) to test whether graphomotor skill is distinct from manual dexterity. Given that the model fit in previous research was adequate, this evidence supports the idea of a single‐factor FMS as per shared processing. However, too few FMS subdomains were likely included to test for a three‐factor solution proposed by functionalism.

Three studies have used a larger number of FMS subdomains/tests, with the first two being conducted on adults. Seashore et al. (1940) administered 21 FMS tests, covering reaction times, tapping rates, high‐speed coordination, postural steadiness, and assembly tests. Consistent with functionalism, they found evidence for six factors, three of which bear overlap to the definitions employed here, namely: (a) finger‐hand speed, (b) forearm and hand speed, (c) manipulation of spatial relations, and others that might not be strictly FMS, specifically (d) reaction time, (e) steadiness, and (f) residual variables.

Again, in a sample of adults, Fleishman and Hempel (1954) found evidence of similar FMS factors to those discussed here. Four hundred airmen completed 15 widely used tests, which included six well‐known tasks (pegboard, block placement, hole punching, weaving, tapping, and tracing). Consistent with a one‐factor solution, one of their models (“centroid”) appeared to show that all tasks loaded on one general FMS factor (loadings > 0.42, split median = 60). However, using a different method, they preferred a five‐factor model, with their labels being finger dexterity, manual dexterity, wrist‐finger speed, aiming, and positioning. Examining the loadings more closely, it seems curious that many variants of the pegboard task loaded on separate factors. Perhaps, had more modern methods of factor analysis been available to them, different model specifications would have been obtained. However, these findings of five to six factors in older samples would appear to support the idea of developmental functionalism. Although adult samples provide insight into the factorial structure in this age group, due to potential developmental effects, it cannot be assumed that the factors apply to children.

In a study of 78 preschool and kindergarten children, Martzog et al. (2019) argued, based on the literature, that a three‐factor structure containing dexterity, speed, and graphomotor skills was the theoretically and empirically most parsimonious. A factor analysis of nine different tests, three in each domain, confirmed this structure. Interestingly, this three‐factor structure in these young preschool children speaks for functionalism and less for shared processing or developmental approaches. However, due to this study's small preschool sample size, replication and extension to older children are needed.

Finally, previous research has not taken account of gender differences in FMS and how this might affect the corresponding factor structure. In meta‐analyses, gender differences in some general motor skills have been found, although findings are somewhat inconsistent as to which skills favor boys versus girls and when (Ikeda and Aoyagi 2008; Thomas and French 1985; Zheng et al. 2022). Turning more specifically to FMS, a clearer pattern of differences emerges, with girls generally showing advantages for dexterity and graphomotor/writing skills (Maurer 2024; Tabatabaey‐Mashadi et al. 2015; Vlachos et al. 2014). Moreover, in addition to gender differences in graphomotor and dexterity favoring girls, Bondi et al. (2022) observed that from grades 1 to 2 (ages 6–8), the structure of FMS was different and changed differently for boys and girls across time. Accordingly, due to the preliminary nature of current research, explorative analyses looking at how gender might affect the factor structure across time are required.

## 4 Current Study

Previous research has identified FMS as an important factor in child development (Gandotra et al. 2021; Strooband et al. 2020; Voyer and Jansen 2017). However, there is little conceptual or empirical agreement as to how best to define, conceptualize, and measure FMS. Researchers currently use a heterogeneous range of terms, measures, and underlying constructs, which is not only potentially confusing but could be confounding attempts to investigate links between FMS and child learning and development. Several theories make different predictions about the FMS construct, from being unitary (i.e., shared processing), differentiated (i.e., functionalism), or assuming developmental changes. However, only a few studies have tested the factor structure of FMS directly, and none have done so with a developmental hypothesis in mind—particularly around the transition to school when FMS might be expected to functionally differentiate. Accordingly, these studies have limitations with respect to the current paper's aims, such as (a) employing parent/teacher report measures (Kim et al. 2015; Lin et al. 2014), (b) having small sample sizes (Martzog et al. 2019), (c) using a limited number of FMS tests (Michel and Gado 2022; Okuda et al. 2019; Schulz et al. 2011), (d) not examining a range of sample ages, (e) containing only adult samples (Fleishman and Hempel 1954; Seashore et al. 1940), and (f) needing further exploration of the role of gender (Bondi et al. 2022).

Accordingly, we recruited two large cohorts of 6 year old kindergarten and 8 year old grade 2 children, tested them twice 1 year apart to capture developmental differences on nine key aspects of FMS derived from established measures to test for functional differences. Therefore, we could compare a single‐factor against a three‐factor model in relation to development across a key transition into academic learning. To investigate this, we adopt a stringent approach to avoid overfitting the model to theoretical preconceptions. We randomly divide the cohort at study entry into two groups and run an exploratory factor analysis on one half and then a confirmatory on the second half. Then, the obtained model structure is tested on the full samples 1 year later. The only previous study (Martzog et al. 2019) conducted on children that employed a similar number of behavioral measures of FMS found evidence for a three‐factor solution. Hence, we hypothesize that the best‐fitting model will contain dexterity, graphomotor, and speed‐FMS factors.

We also include tests of developmental effects by examining model fit and factor loading across the cohorts as a function of age, again by conducting explorative and confirmatory analyses on split samples in kindergarten and grade 2. Shared‐processing theories would predict a unitary FMS construct, or a general factor, in all age ranges studied here. Functionalism would predict a differentiated three‐factor model in all age ranges due to specialization occurring in home environments and educational settings (Caçola et al. 2011; Suggate et al. 2016). Concerning developmental effects, FMS might become more unitary with age as FMS skills become integrated, according to shared processes (i.e., developmental increases in shared processing). Conversely, FMS might become more differentiated with age as children's FMS specialize (i.e., developmental increases in functionalism).

Finally, we conducted explorative analyses testing the role of gender. Consistent with previous research, it seemed reasonable to anticipate greater dexterity and graphomotor skills for girls (Bondi et al. 2022; Tabatabaey‐Mashadi et al. 2015; Vlachos et al. 2014). Due to the lack of previous research (cf. Bondi et al. 2022), the analysis concerning whether the FMS factor structure changes differently over time for girls versus boys was explorative.

## 5 Methods

### 5.1 Participants

Two cohorts of children participated in this study. The first comprised 240 kindergarten children, of which 48.6% were female, one child diverse, and the rest male. They were aged 6 years and 1.41 months (SD = 5 months) and 75.3% of the children spoke exclusively German at home. In terms of country of origin, 76.7% (parent 1) and 71.7% (parent 2) of the children's parents were born in Germany, with the remainder being fairly evenly split across 22 different countries, and 88.3% of the children themselves were born in Germany. Of the parents, 37.1% had attained a university degree or equivalent, with the rest having a university entrance qualification (22.7%), an upper secondary school qualification (20.9%), a lower secondary school qualification (15.8%), as their highest educational attainment, or no school qualification (2.5%). In terms of school qualifications for adults aged 25 to 35 years, this approximately corresponds to the general population for adults in this age range, *χ* ^2^(3) = 1.71, *p* = 0.634 (Federal Office for Political Education 2025). Most children were tested in the years 2022 to 2023, however, a small cohort in years 2020 to 2021 due to pandemic disruptions.

The older cohort comprised 310 children, of which 50.5% were female, aged 8 years and 4 months (SD = 6 months), and 68.1% spoke exclusively German at home. In terms of country of origin, 72.9% (parent 1) and 69.0% (parent 2) of the parents were born in Germany, with the remainder coming from 38 different countries, and 89.0% of the children were born in Germany. In terms of highest educational qualification, this was: a university degree (40.0%), a university entrance qualification (20.0%), an upper secondary school qualification (23.0%), a lower secondary school qualification (14.0%), or no school qualification (2.0%). Again, this approximately corresponds to the general population for adults in this age range, *χ* ^2^(3) = 1.86, *p* = 0.602 (Federal Office for Political Education 2025).

### 5.2 Measures

Before conducting the motor tests, we first determined children's dominant hand by directly asking them in the older cohort. For the younger children, we observed the hand that the children used when writing, and then asked them whether this was the hand they always used for writing. As part of a larger longitudinal study, further measures not reported here were included.

5.2.1

A total of 10 measures of FMS were used, nine in each cohort, to capture a range of dexterity, graphomotor, and speed behaviors.

#### 5.2.2 Dexterity

Four measures of dexterity were drawn partly from the Movement‐ABC (Petermann et al. 2011), with some critical changes. First, we wanted a larger behavioral sample than that provided by the Movement‐ABC, so we used a larger pegboard with more holes/pegs. Second, to ensure comparability across all cohorts, we administered the same number of trials for each test and conducted the same tests across all participants. Third, we also administered the weaving task to preschool children, even though this is usually given to school‐age children. The key reasons for doing this were to provide direct comparability with the older children and to avoid floor effects because the Movement‐ABC was originally designed as a screening measure.

##### 5.2.2.1 Coin Posting

The coin‐posting task from the Movement‐ABC age group 1 was used to measure dexterity in all children. Twelve yellow coins and a small blue box with a slit in the top were laid out on a nonslip desk pad. Children were instructed to hold the box with one hand while posting the coins, one at a time, through the slot. The task was administered twice to each hand, and the time to post all coins was recorded and summed across all four trials. Internal consistency was excellent, in kindergarten through grade 3, *α*_cr_ = 0.89, 0.85, 0.84, and 0.86, respectively.

##### 5.2.2.2 Bead‐Threading

The bead‐threading task from the Movement‐ABC age group 2 was administered to all participants (Petermann et al. 2011). Children are given 12 beads, placed out in front of them on a nonslip desk pad, with a red shoelace‐like thread. After a demonstration, children are required to take the thread in one hand and pick up and place the beads on the thread, one at a time. Completion time was recorded, and two trials were conducted, which were summed together. Internal consistency was excellent, in kindergarten through grade 3, *α*_cr_ = 0.87, 0.84, 0.87, and 0.84, respectively.

##### 5.2.2.3 Weaving

The weaving task from the Movement‐ABC (Petermann et al. 2011) was also used here and administered to all participants. Again, a nonslip desk pad was used, upon which a yellow plastic rectangle with eight holes in a row was placed, along with the red shoelace‐like thread. Children were asked to thread the holes in a sequential, weaving motion without missing any holes. Time to complete all eight holes was recorded and two trials were administered, with the total score representing the sum of both trials. Internal consistency was very good; in kindergarten through grade 3, *α*_cr_ = 0.76, 0.78, 0.73, and 0.77, respectively.

##### 5.2.2.4 Pegboard

The Purdue pegboard test (Tiffin and Asher 1948) was used as a measure of manual dexterity. The pegboard task has two parallel columns of 24 holes each and trays with pegs, which are to be inserted one at a time from left to right for the right hand and from right to left for the left hand in a second trial. Time for 10 and all 24 pegs was taken for each side and summed together. Internal consistency was very good, in kindergarten through grade 3, *α*_cr_ = 0.79, 0.78, 0.70, and 0.79, respectively.

#### 5.2.3 Graphomotor Skills

Four measures of graphomotor skills were included that required using a (pencil to create forms and shapes). Two measures were taken from the Beery‐Buktenica Developmental Test of Visual‐Motor Integration (Beery and Buktenica 1982), one was adapted from previous work (Stoeger et al. 2008), and the fourth was created for the purpose of this study. However, only one letter copying task was included in each cohort due to time and developmental constraints, beginning with Greek letters in the younger children and moving over to copying entire nonwords in the older cohort.

##### 5.2.3.1 Beery—Tracing

For the tracing task, children are given a series of forms increasing in complexity, in which they are asked to draw a line following the contours but without touching the guiding lines (Beery and Buktenica 1982). Children are given 5 min to complete as many of 27 forms as possible, plus three were automatically awarded to all children for following instructions, giving a maximum of 30 points.

##### 5.2.3.2 Beery—Forms

The Beery Form copying task requires children to copy as many geometric forms, increasing in complexity, as they can within 5 min (Beery and Buktenica 1982). One point is provided per completely and correctly copied form, with a maximum score of 30.

##### 5.2.3.3 Greek Letter Copying

Based on a procedure used by Stoeger et al. (2013), children were presented with the Greek alphabet and asked to copy as many of the forms as they could within 3 min. In total, 26 letters were presented, and these were rated for completeness and correct form. Due to time constraints, this test was not administered to the older cohort. Scores for 20 children were rated months after the testing session by two independent raters, achieving excellent agreement of 93%, *κ* = 0.84.

##### 5.2.3.4 Nonword Copying

Children's graphomotor skills were also tested using a pseudoword copying task. Pseudowords were selected instead of real words because we wanted to partial out effects for word familiarity, which might facilitate copying performance. A piece of paper was placed in front of children, and they were asked to begin copying the words, paying attention to upper and lower‐case letters. The score was the correct number of words copied in 1 min.

#### 5.2.4 Speed

Two measures of speed were used, a rabbit and a fire extinguisher task.

##### 5.2.4.1 Rabbit Tapping

For the rabbit task, children are to help a rabbit run across a screen to a carrot by pressing a key as quickly as possible. Children were instructed to rest their hand on a hand rest and not raise it until they had finished the task, to standardize the procedure. Two trials per child were conducted, one with each hand, and the number of key presses within 15 s was recorded by the computer and summed across both trials. Internal consistency was excellent, in kindergarten through grade 3, *α*_cr_ = 0.77, 0.79, 0.82, and 0.83, respectively.

##### 5.2.4.2 Fire Extinguisher

For the fire extinguisher task (Martzog et al. 2019), children were shown a picture of a burning house and were asked to put this fire out with “rain drops” by putting as many dots as possible within 10 s on the house, using a blue felt pen. The responses were recorded using an audio recorder, and one trial per hand was conducted and summed together. Internal consistency was excellent; in kindergarten through grade 3, α_cr_ = 0.72, 0.75, 0.68, and 0.74, respectively.

### 5.3 Procedure

Ethics approval for this research was provided by the University ethics committee and according to state regulations, separate approval was then received from the Ministry of Education for conducting research in schools. We then approached educational institutions via their management personnel, and if invited to do so, briefed them and responsible staff as to the study aims and procedures, in accordance with their institutional preferences. Next, we invited all parents of children in the target age range. No participants were excluded based on a pre‐existing condition or disability.

Cohort 1 children were recruited from local kindergartens and schools in and around a small city in Germany. In Germany, children generally attend kindergartens, which are separate institutions with a nonacademic, often play‐based curriculum, until the year in which they turn 7. Accordingly, we followed the 5‐ and 6 year old children from these smaller kindergartens into the schools that they attended in grade 1, 1 year later. Testing took place on site by trained research assistants under the leadership of the second author, usually in a small room in the educational institution. At each time point, 12 months apart, testing was divided into two sessions of approximately 30 min to reduce participant fatigue. Additionally, to maintain motivation, we varied the task order, interspersing motor tasks with reading and writing measures (letter naming, word reading, letter writing, word writing, phonemic awareness, reading fluency and comprehension for the older children), the latter being collected as part of a larger study but not reported here. For the school‐age children in cohort 2, the Beery tests were administered in group settings according to test instructions in a single session. Task order was not counter‐balanced.

### 5.4 Analyses

To test our hypothesis that a three‐factor model of FMS would best explain the data in a stringent manner, while accounting for the notion that developmental and cohort differences existed, we adopted a stringent approach, combining explorative and confirmatory factor analyses using a split‐sample approach (Lorenzo‐Seva 2022). To establish cohort comparability while allowing for potential developmental differences, both exploratory and confirmatory factor analyses on each cohort were conducted at time 1 (i.e., kindergarten and grade 2). We did this by randomly splitting the sample into two using the SPSS “random sample” function. On the one half of each sample, we conducted exploratory factor analyses using oblimin rotation to allow factors to correlate with each other (Tabachnick and Fidell 2007). On the second half of the sample, and on the full sample in grade 1 and 3, we conducted confirmatory factor analyses in R using the lavaan package (Rosseel 2012), testing for three factors (functionalism) and comparing this against one‐factor models (shared‐processing), and using full information likelihood to account for missing data. Further, by virtue of the exploratory factor analysis, and the confirmatory factor analyses comparing one versus three factor solutions, we could test for developmental differences. In total, for the main analyses, two exploratory (kindergarten, grade 2) and four confirmatory models were run at each time point from kindergarten to grade 3 to test for a three‐factor solution (i.e., dexterity, graphomotor, and speed FMS). Finally, gender differences were explored by conducting independent sample t‐tests across all FMS measures in all cohorts. This was followed by exploratory factor analyses in each cohort, separated by gender.

## 6 Results

Data were first screened for outliers, which were winsorized at four standard deviations above the mean. This resulted in a small percentage of data being winsorized (0.28%). We chose this high threshold to retain as much natural variance in the data as possible and yet result in distributions not violating statistical assumptions. Descriptives were screened for skew and kurtosis, which revealed almost always minimal skewness and peakedness. The descriptive statistics, also broken down separately for boys and girls, appear in Tables 1 and 2. The findings in Tables 1 and 2 indicate an improvement in the task completion times for the dexterity measures with increasing age, and also an increasing performance score on the number of forms copied (Beery and letter/word copying) and taps (Rabbit and Fire task). Correlation coefficients for all of the tests as a function of cohort were calculated, and these appear in the Tables (S1–S4). As can be seen in these tables, most tasks show moderate to strong correlations with each other, perhaps less so for some of the graphomotor tasks and the tapping measures.

**TABLE 1:** Descriptive statistics and correlations for the kindergarten to grade 1 cohort.

Time	Measure	Combined			Boys			Girls			*t*	*p*	*d*	Advantage
		*M*	SD	*n*	*M*	SD	*n*	*M*	SD	*n*				
Kindergarten	Coin posting	97.245	16.674	219	96.684	17.128	112	97.832	16.244	107	−0.508	0.612	−0.069	
	Bead threading	93.635	20.748	219	97.616	21.016	112	89.467	19.713	107	2.956	0.003	0.393	Girls
	Weaving	82.749	26.504	219	83.786	24.252	112	81.664	28.749	107	0.591	0.555	0.080	
	Pegboard	130.037	18.913	218	130.514	19.397	111	129.542	18.475	107	0.378	0.706	0.051	
	Beery tracing	17.009	2.976	220	16.263	2.875	114	17.811	2.885	106	−3.984	0.000	−0.520	Girls
	Beery forms	16.237	2.312	219	16.088	2.336	113	16.396	2.287	106	−0.984	0.326	−0.133	
	Greek letters	6.127	4.127	220	5.175	3.895	114	7.151	4.142	106	−3.646	< 0.000	−0.479	Girls
	Rabbit speed	105.316	19.423	212	105.793	19.073	111	104.792	19.882	101	0.374	0.709	0.052	
	Fire task	75.155	10.726	213	75.804	10.995	107	74.500	10.458	106	0.887	0.376	0.122	
Grade 1	Coin posting	87.277	12.319	199	86.812	12.223	100	87.747	12.460	99	−0.535	0.593	−0.076	
	Bead threading	79.071	15.617	200	81.764	17.214	101	76.323	13.334	99	2.496	0.013	0.348	Girls
	Weaving	65.300	18.287	200	67.613	20.391	101	62.939	15.610	99	1.818	0.256	0.071	
	Pegboard	115.780	14.412	196	115.652	14.381	98	115.908	14.516	98	−0.124	0.901	−0.018	
	Beery tracing	18.353	3.077	201	18.137	3.252	102	18.576	2.886	99	−1.010	0.314	−0.142	
	Beery forms	18.025	2.058	200	18.000	2.101	102	18.051	2.022	98	−0.175	0.861	−0.025	
	Greek letters	10.041	3.766	193	9.697	3.829	99	10.404	3.685	94	−1.306	0.193	−0.188	
	Rabbit speed	112.780	17.054	200	115.644	16.777	101	109.859	16.920	99	2.428	0.016	0.339	Boys
	Fire task	83.918	10.318	184	84.245	10.148	94	83.578	10.538	90	0.437	0.662	0.065	

**TABLE 2:** Descriptive statistics and correlations for grade 2.

Time	Measure	Combined			Boys			Girls			*t*	*p*	*d*	Advantage
		*M*	SD	*n*	*M*	SD	*n*	*M*	SD	*n*				
Grade 2	Coin posting	80.714	11.610	303	79.531	11.562	153	81.920	11.573	150	1.798	0.073	−0.206	
	Bead threading	73.375	14.819	304	70.336	12.029	153	76.454	16.672	151	3.673	< 0.000	−0.413	Boys
	Weaving	56.753	14.991	304	54.246	14.110	153	59.293	15.469	151	2.973	0.003	−0.337	Boys
	Pegboard	108.776	15.385	304	107.699	15.595	153	109.867	15.142	151	1.230	0.220	−0.141	
	Beery tracing	18.146	2.786	302	18.555	2.658	155	17.714	2.860	147	−2.647	0.009	0.302	Boys
	Beery forms	19.249	2.565	297	19.399	2.509	153	19.090	2.623	144	−1.036	0.301	0.120	
	Nonword writing	8.429	3.182	280	9.062	3.127	145	7.748	3.109	135	−3.523	< 0.000	0.413	Boys
	Rabbit speed	120.487	20.262	304	118.843	21.085	153	122.152	19.320	151	1.426	0.155	−0.163	
	Fire task	87.526	11.592	272	87.275	11.522	135	87.774	11.697	137	0.354	0.723	−0.043	
Grade 3	Coin posting	75.511	10.103	306	75.006	9.763	154	76.022	10.443	152	0.879	0.380	−0.100	
	Bead threading	63.599	10.269	307	61.044	9.278	155	66.204	10.601	152	4.541	< 0.000	−0.503	Boys
	Weaving	49.209	12.659	306	45.978	11.042	154	52.482	13.369	152	4.642	< 0.000	−0.514	Boys
	Pegboard	100.142	12.462	306	98.703	12.266	154	101.599	12.529	152	2.043	0.042	−0.232	Boys
	Beery tracing	19.787	3.060	305	20.497	2.940	153	19.072	3.021	152	−4.172	< 0.000	0.465	Boys
	Beery forms	19.890	2.493	292	20.286	2.499	147	19.490	2.430	145	−2.759	0.006	0.319	Boys
	Nonword writing	11.539	3.304	306	12.058	3.502	154	11.013	3.012	152	−2.798	0.005	0.316	Boys
	Rabbit speed	130.326	18.533	307	129.497	19.455	155	131.171	17.567	152	0.791	0.430	−0.090	
	Fire task	95.376	11.076	298	95.196	11.188	148	95.553	10.999	150	0.278	0.781	−0.032	

### 6.1 Explorative Factor Analyses

The first explorative factor analysis was conducted on a random sample of the kindergarten cohort (*n* = 118) and included the nine FMS measures. Oblimin rotation was used to allow for the possibility that any FMS factors correlated with each other. The corresponding scree plots are presented in Figure S1 and using an Eigen value cutoff of 1, three factors were obtained, which explained 65.46% of the variance. The same procedure was followed for the grade 2 sample (*n* = 161), which also resulted in three factors explaining 65.47% of the variance. Factor loadings for both models are displayed in Table 3. As Table 3 shows, the nine tests loaded in an expected manner onto their respective factors, which correspond to dexterity, speed, and graphomotor skills. However, the loadings were less clear cut in grade 2, with the dexterity tasks showing small to moderate loadings onto the speed factor (Factor 2) and also the Beery tasks loading onto the dexterity factor.

**TABLE 3:** Standardized factor loadings for explorative factor analysis on part samples.

Variable	Kindergarten			Grade 2		
	Factor 1	Factor 2	Factor 3	Factor 1	Factor 2	Factor 3
Dexterity						
Bead threading	**0.722**	−0.002	−0.013	**0.852**	−0.223	−0.117
Coins posting	**0.902**	−0.019	0.164	**0.796**	−**0.365**	−0.003
Weaving	**0.570**	0.087	−0.225	**0.708**	−0.174	−0.188
Pegboard	**0.788**	−0.066	−0.102	**0.855**	−0.309	−0.005
Speed						
Rabbit task	0.004	**0.844**	−0.040	−0.178	**0.901**	0.062
Fire task	−0.023	**0.833**	0.065	−0.382	**0.875**	−0.092
Graphomotor						
Beery tracing	0.077	0.018	**0.854**	−0.251	0.169	**0.744**
Beery form	−0.144	−0.051	**0.742**	**−0.421**	0.019	**0.584**
Greek letters	−0.012	0.053	**0.823**			
Nonword				**−0.355**	0.216	**−0.604**

*Note:* *N* = 118 and 161, respectively. The extraction method was principal component with an oblimin (Kaiser Normalization) rotation. Factor loadings greater than 0.32 are in bold. Greek letters administered to children in Kindergarten and Grade 1 only and nonword writing in grade 2 and 3 only.

### 6.2 Confirmatory Factor Analyses

Next confirmatory factor analyses were conducted, testing the model found in the exploratory factor analysis on the second half of the kindergarten and grade 2 samples, and then on the entire grade 1 and 3 samples. Additionally, to test the hypothesis that the three‐factor model would better fit the data than the single‐factor model, the latter were also included and contrasted. The different models and fit indices appear in Table 4.

**TABLE 4:** Model parameters comparing one‐, two‐, and three‐factor confirmatory models for the kindergarten through grade 3 cohorts.

Model	df	*X* ^2^	*p*	CFI	TLI	RMSEA	SRMR	AIC	BIC	aBIC
Kindergarten (*n* = 122)										
One‐factor model	27	59.600	< 0.001	0.874	0.832	0.100	0.071	7619.048	7694.534	7609.169
Three‐factor model	24	26.581	< 0.001	0.990	0.985	0.030	0.044	7592.028	7675.902	7581.052
Grade 1 (*n* = 240)										
One‐factor model	27	124.037	< 0.001	0.791	0.722	0.129	0.088	13179.519	13270.526	13184.968
Three‐factor model	24	45.699	< 0.001	0.953	0.930	0.065	0.047	13107.180	13208.299	13113.236
Grade 2 (*n* = 147)										
One‐factor model	27	90.261	< 0.001	0.781	0.708	0.127	0.091	8775.965	8856.522	8771.082
Three‐factor model	24	35.551	< 0.001	0.960	0.940	0.057	0.059	8727.255	8816.763	8721.830
Grade 3 (*n* = 310)										
One‐factor model	27	168.840	< 0.001	0.797	0.729	0.130	0.079	18495.431	18596.318	18510.685
Three‐factor model	24	80.593	< 0.001	0.919	0.879	0.089	0.050	18413.184	18525.281	18430.132

*Note:* The kindergarten and grade 2 samples are randomly selected half samples.

Abbreviations: AIC = Akaike information criterion, BIC = Bayes information criterion, BIC = sample‐size‐adjusted BIC, CFI = comparative fit index, RMSEA = root‐mean‐square estimation, SRMR = standardized mean‐square residual, TLI = Tucker–Lewis index.

As can be seen in Table 4, the three‐factor model was a superior fit compared to the one‐factor models at every age. The three‐factor models showed good data fit, with CFIs usually above 0.95 and RMSEAs of around 0.05. The three‐factor model in grade 3 appeared to fit the data slightly less well (CFI = 0.92 and RMSEA = 0.09); it still fit much better than a baseline or one‐factor model. In Table 5, the factor loadings for the four three‐factor models are presented. It is evident in Table 5 that the factor loadings from the exploratory factor analyses are mirrored and replicated for all cohorts in the confirmatory factor analyses.

**TABLE 5:** Standardized factor loadings for three‐factor confirmatory models of fine motor skills from kindergarten to grade 3.

Variable	Kindergarten	Grade 1	Grade 2	Grade 3
Dexterity				
Bead threading	0.765	0.693	0.690	0.711
Coin posting	0.708	0.773	0.837	0.738
Weaving	0.607	0.609	0.478	0.588
Pegboard	0.752	0.782	0.790	0.763
Speed				
Rabbit task	0.512	0.508	0.717	0.571
Fire task	0.631	0.757	0.791	0.961
Graphomotor				
Beery tracing	0.588	0.742	0.759	0.491
Beery form	0.680	0.595	0.507	0.389
Nonword	—	—	−0.033	0.456
Greek letters	0.755	0.555	—	—

*Note:* Greek letters administered to children in Kindergarten and grade 1 only and nonword writing in grades 2 and 3 only.

Given the lesser model fit in grade 3 and the increased double loadings observed in the grade 2 explorative analyses, an ad‐hoc explorative factor analysis was run on the entire grade 3 sample. Although this also favored a three‐factor solution with an Eigenvalue cut score of 1, there were more ambiguous factor loadings again. For instance, bead threading (*λ* = 0.50) and weaving (*λ* = 0.66) loaded strongly on the graphomotor factors as well as on the dexterity factor, nonword writing (*λ* = 0.71) and tapping (*λ* = 0.30) on dexterity.

### 6.3 Exploring the Role of Gender

There were gender differences in performance on a number of FMS tasks in both cohorts, as can be seen in Tables 1 and 2, with girls initially having better dexterity and graphomotor skills, but with boys being better in tapping in the younger cohort and better in most FMS tasks in the older cohort. Post hoc *t*‐tests confirmed that the boys and girls were equivalent in age in both cohorts. The exploratory factor analyses (see Tables 6 and 7) found a three‐factor solution for both boys and girls in the older cohorts. In the younger cohorts, one factor analysis for 5‐ to 6 year old boys and one for 6 to 7 year old girls marginally suggested a two‐factor solution according to the Eigenvalue cut score (= 1.0). In the two‐factor solutions, the dexterity and graphomotor tasks tended to load on the same factor. For girls in grade 1, the dexterity tasks loaded moderately (around 0.5) onto the speed factor. Generally, the highest loadings were obtained for the dexterity tasks on the dexterity factor (around 0.75).

**TABLE 6:** Standardized factor loadings for explorative factor analysis as a function of gender for the younger cohort.

Cohort	Variable	Boys			Girls		
		Factor 1	Factor 2	Factor 3	Factor 1	Factor 2	Factor 3
Kinderg.	Dexterity						
	Bead thread	**0.723**	0.234	—	**0.799**	−0.212	−0.154
	Coins posting	**0.743**	0.003	—	**0.824**	−0.190	−0.057
	Weaving	**0.704**	−0.080	—	**0.653**	0.172	−0.450
	Pegboard	**0.756**	−0.221	—	**0.845**	−0.157	−0.307
	Speed						
	Rabbit task	0.043	**0.808**	—	−0.214	**0.735**	−0.037
	Fire task	−0.111	**0.827**	—	−0.144	**0.757**	0.278
	Graphomotor						
	Beery tracing	**−0.596**	−0.056	—	−0.222	0.125	**0.794**
	Beery form	**−0.748**	−0.033	—	−0.217	0.215	**0.806**
	Greek letters	**−0.595**	0.237	—	−0.107	**0.532**	0.**409**
Grade 1	Dexterity						
	Bead thread.	**0.801**	0.132	−0.242	**0.688**	**0.449**	—
	Coins posting	**0.762**	0.186	**−0.345**	**0.694**	**0.581**	—
	Weaving	**0.767**	0.027	−0.262	**0.664**	0.195	—
	Pegboard	**0.798**	0.277	−0.261	**0.707**	**0.546**	—
	Speed						
	Rabbit task	−0.077	**−0.864**	−0.009	−0.009	**−0.657**	—
	Fire task	−0.233	**−0.826**	0.066	−0.165	**−0.743**	—
	Graphomotor						
	Beery tracing	**−0.375**	−0.070	**0.828**	**−0.713**	−0.007	—
	Beery form	−0.119	0.025	**0.853**	**−0.627**	0.210	—
	Greek letters	**−0.383**	−0.059	**0.613**	**−0.715**	−0.030	—

*Note:* The extraction method was principal component with an oblimin (Kaiser Normalization) rotation. Factor loadings greater than 0.32 are in bold.

Abbreviation: Kinderg., kindergarten.

**TABLE 7:** Standardized factor loadings for explorative factor analysis as a function of gender for the older cohort.

Cohort	Variable	Boys			Girls		
		Factor 1	Factor 2	Factor 3	Factor 1	Factor 2	Factor 3
Grade2	Dexterity						
	Bead threading	**0.805**	0.158	−0.262	**0.844**	**−0.393**	−0.059
	Coins posting	**0.811**	0.063	−0.085	**0.839**	**−0.353**	−0.201
	Weaving	**0.714**	0.107	−0.168	**0.654**	−0.025	−0.312
	Pegboard	**0.855**	0.133	−0.275	**0.830**	**−0.373**	−0.087
	Speed						
	Rabbit task	−0.145	0.008	**0.879**	−0.286	**0.857**	0.215
	Fire task	−0.308	0.012	**0.858**	**−0.369**	**0.845**	0.028
	Graphomotor						
	Beery tracing	−0.254	**−0.767**	0.101	−0.200	0.185	**0.764**
	Beery form	**−0.322**	**−0.671**	−0.143	**−0.358**	0.045	**0.698**
	Nonword	−0.163	**0.645**	0.032	−0.285	**0.565**	**−0.380**
Grade 3	Dexterity	**0.733**	−0.295	0.211	**0.827**	**−0.407**	**−0.380**
	Bead threading	**0.779**	−0.172	0.003	**0.728**	−0.269	**−0.395**
	Coins posting	**0.688**	−0.109	−0.236	**0.543**	−0.145	**−0.666**
	Weaving	**0.762**	−0.237	0.163	**0.823**	−0.279	**−0.399**
	Pegboard						
	Speed						
	Rabbit task	−0.078	**0.861**	−0.035	−0.262	**0.903**	0.072
	Fire task	−0.291	**0.852**	0.055	**−0.374**	**0.858**	0.292
	Graphomotor						
	Beery tracing	**−0.616**	0.108	**0.335**	−0.286	0.118	**0.745**
	Beery form	**−0.338**	**0.325**	**0.720**	−0.198	0.233	**0.817**
	Nonword	**−0.454**	**0.338**	**−0.648**	**−0.656**	0.208	0.015

*Note:* The extraction method was principal component analysis with an oblimin (Kaiser Normalization) rotation. Factor loadings greater than 0.32 are in bold.

## 7 Discussion

Recent years have seen an exponential growth in studies in developmental psychology and education investigating how FMS relate to cognitive development and academic performance (Cameron et al. 2012; Grissmer et al. 2010; Luo et al. 2007; Suggate et al. 2023). However, a glance over this literature reveals problematic differences in the definition, measurement, and conceptualization of FMS. Accordingly, in the current study, we derived and tested a series of theoretically plausible models of the structure of FMS, which—despite the large number of studies and the centrality of this question for measuring and researching FMS—have received little empirical attention.

We found strong—but not unequivocal—evidence for the three‐factor structure of FMS containing the constructs of dexterity, speed, and graphomotor skills, as predicted by functionalism. A three‐factor structure emerged in exploratory factor analyses in the 5‐ to 6 year old kindergarten and eight‐ to 9 year old grade 2 children, with clear loadings in kindergarten and adequate loadings in grade 2 on the corresponding dexterity, graphomotor, and speed FMS factors. This factor structure was then replicated in confirmatory factor analyses on independent samples in kindergarten through to grade 3. Our study replicates the three‐factor structure found in a small sample of 4‐ to 6 year old children (Martzog et al. 2019) but extends these findings through to age nine, or grade 3. Moreover, the exploratory factor analyses did not provide strong evidence to suggest that the factor structure for boys and girls differed greatly. This three‐factor structure would appear to make good sense given the age of children studied here, with graphomotor skills being learned in kindergarten in preparation for formal schooling.

The evidence was not strong in terms of a pure shared‐processing or embodiment account. No single‐factor models emerged as best fitting in the exploratory or confirmatory factor analyses. Thus, in contrast to previous work arguing for a single factor (e.g., Hands et al. 2018) or prominent tests of FMS (e.g., the Movement ABC, Petermann et al. 2011), it does not seem that treating FMS as a unitary construct in kindergarten and primary school is advisable. Indeed, our findings likely differ from previous research supporting a single or general FMS factor precisely because such work generally used the Movement‐ABC (Michel and Gado 2022; Oberer et al. 2017; Schulz et al. 2011), which contains only three FMS sub‐tests representing different constructs, with only one trial feeding into the total score. In contrast, we had a total of 17 trials or scales across the nine FMS tests, including both dominant and nondominant hands (e.g., four coin‐posting, two each of pegboard, weaving, threading, and tapping trials). This likely gave us a much larger behavioral sample to test the underlying constructs.

Although not supporting a purely embodied account, the exploratory factor analyses in the 8 year old grade 2 children and, in particular, an ad‐hoc explorative factor analysis in 9 year olds suggested a developmental shift toward a less specialized and more general factor structure later in development. Specifically, the clear factor structure in kindergarten appeared to progressively weaken across grade 1 and through into grade 3. This observation is important because it suggests that the reduced model fit was due to age, not cohort, because this pattern extended across cohorts (e.g., RMSEA worsened from kindergarten to grade 3: 0.03, 0.06, 0.06, 0.09, respectively). Importantly, the different subtests exhibited good reliability and generally correlated moderately to strongly with each other, suggesting that reliability issues were not the cause of these findings. Therefore, we tentatively conclude that the factor structure began a subtle transition after kindergarten, showing a developmental tendency away from a functionalist to a shared‐processing account. Accordingly, we strongly recommend that future research examine this potential developmental trend by studying the factor structure in similar and older samples.

Findings also yielded data relevant to understanding gender differences in FMS, which have been found previously for FMS and related abilities (Vlachos et al. 2014), such as cube drawing (Lange‐Küttner and Ebersbach 2013; Tabatabaey‐Mashadi et al. 2015). Contextualizing the current findings in this research, it is interesting that both here and in previous studies, the advantage for girls in FMS has generally been found in younger children, up until around age 6 or 7 years old (Bondi et al. 2022; Maurer 2024; Tabatabaey‐Mashadi et al. 2015). Furthermore, two meta‐analyses found a tendency for boys' FMS to increase relative to girls across early childhood until age 6, which was the oldest investigated age group in these studies (Ikeda and Aoyagi 2008; Zheng et al. 2022). Our results suggest that this trend continues into the age range of 7–10.

## 8 Limitations and Future Research

Although the chi‐square statistics indicated a superiority for the three‐factor models over the one‐factor models at each time point, other fit indices (e.g., CFI, RMSEA) indicated that the three‐factor model might still not be optimal. Future research should accordingly continue to investigate the structure in older children. To capture developmental differences, we used two different graphomotor tasks (i.e., Greek letters and nonwords) on the different cohorts. These task differences may have limited comparisons in the factor constructs across cohorts, possibly also leading to changes in the factor structure for the older children.

Furthermore, the current study included two large cohorts and 17 different FMS subtests; hence it is possible that including further measures would have resulted in different factor structures. Candidates here might be finger gnosia, bimanual coordination, or communicative gestures (Beilock and Goldin‐Meadow 2010; Dellatolas et al. 2003; Penner‐Wilger and Anderson 2013). Additionally, the speed and graphomotor factors contained comparatively fewer measures than the dexterity domain, perhaps resulting in a smaller behavioral sample that is then less stable. However, there was consistency across the four different grades, which would suggest that sufficient power to detect factors was available. Given that we did not include measures of gross motor skills, we cannot discount general motor theories (Hands et al. 2018), even though it would logically be expected that if such theories were correct, all FMS domains should also load on one factor.

Our analyses regarding gender revealed interesting differences, particularly in absolute FMS performance, but need to be replicated and extended on a larger sample to investigate possible gender differences in factor structure (e.g., Bondi et al. 2022). Additionally, given that our study comprised two longitudinal cohorts (from kindergarten to grade 1 and from grade 2 to grade 3), conclusions regarding developmental changes in the factor structure are confounded with potential cohort differences.

Theoretically, graphomotor skills are difficult to conceptualize and measure because tasks often implicitly tap cognitive knowledge of stored letter forms (Araújo et al. 2022). We tried to circumvent this difficulty by using unfamiliar forms, letters, words, and symbols as stimuli. However, it is possible that cognitive confounds were present across participants and cohorts due to individual differences in prior knowledge (Bourke et al. 2014). Interestingly, the Greek letter copying task appeared to load more strongly on the graphomotor skills factor than the nonword tasks, perhaps because the latter was more confounded with prior symbol knowledge. If the graphomotor construct is defined to be purely concerned with pencil/pen operation, then it is easy to see how this would be earlier distinct from manual dexterity.

Regarding the graphomotor factor, the nonword copying task in grade 2 did not load strongly with the Beery tasks on that factor. A likely explanation is that children were still grappling with the cognitive demands of copying letters in grade 2, and hence, the test tapped letter and word knowledge in addition to motor skills. However, by grade 3, word reading skills were likely sufficient to allow children to focus more on copying the pseudowords, resulting in a measure that better reflected graphomotor skills. Future research might include a more thorough examination of writing and graphomotor skill tasks. Additionally, we did not explicitly investigate the role of handedness, which might be an interesting venture for future research. Additionally, we included cohorts from kindergarten to grade 3, limiting our conclusions regarding the general structure of FMS across a larger range of ages. Future research could extend this into adolescence and adulthood, perhaps studying how changes accompany environmental specialization as participants enter different professions (e.g., dentist, typist, builder, psychologist). Finally, given the presumed effects of schooling on FMS and particularly graphomotor skills, the factor structure found here might vary in different educational systems with different starting ages and curricula.

## 9 Implications

However, key implications from the current study concern research and practice. Beginning with research, it would seem crucial that clarity as to the conceptualization of FMS as a construct is key. Arguably, findings on links between FMS and academic and cognitive skills have been mixed, with many null findings interspersed with moderate effects (e.g., Cameron et al. 2012; Grissmer et al. 2010; Suggate et al. 2018; Suggate et al. 2019; Wassenberg et al. 2005). Perhaps part of the contradictory evidence is due to an inconsistent conceptualization of FMS, with this strewn across multiple measures and labels (e.g., visuo‐motor, hand–eye coordination, manual skill, etc.). Such diffusion also compounds the identification of theoretical pathways, preventing researchers from specifying constructs and mechanisms through which FMS influence development. Importantly, although our findings from over a dozen subtests generally support three factors, this does not mean that future research needs to include such an exhaustive battery. Instead, depending on the research question, future research could likely include a more minimalist battery. One suggestion would be the pegboard task for dexterity, the Beery tracing for graphomotor skill, and any tapping task with a 30 s behavioral sample to assess speed‐FMS.

Turning to practical implications, these can be divided into diagnosis and education/therapy. Beginning with diagnosis, a clear understanding of the factor structure of FMS allows the development of targeted and accurate measures to inform not only motor development deficits but also conceptions of school readiness (Duncan et al. 2007; Grissmer et al. 2010). In terms of educational significance, it appears that there is generalizability between subcategories of FMS. Accordingly, interventions to improve FMS may focus on specific skills (e.g., handwriting using graphomotor training), but our findings might also indicate that broader is better. Thereby, a rich range of opportunities to physically interact with the environment differentiates general FMS into subdomains in kindergarten and primary school (Fisher et al. 2005; Fitzpatrick et al. 2012; Hadders‐Algra 2010; Mavilidi et al. 2018).

## 10 Summary

An old adage is that carpenters are only as good as their tools; likewise, accurate research on FMS requires accurate measures, which clearly encompasses including the right tests in the right domains—and likely also in light of the current findings at the right time in child development. The current study indicates that viewing FMS as a differentiated construct in kindergarten through to grade 3, encompassing dexterity, graphomotor, and speed‐FMS, represents an appropriate structure—seemingly justifiably cutting FMS fine.

## Supporting information


**Table S1.** Correlations for the younger sample at time 1 (kindergarten).
**Table S2.** Correlations for the younger sample at time 2 (grade 1).
**Table S3.** Correlations for the older sample at time 1 (grade 2).
**Table S4.** Correlations for older sample at time 2 (grade 3).

**Figure S1.** Screen plot for the explorative factor analyses for the kindergarten (above) and grade 2 (below) randomly split samples.

Supinfo

## Data Availability

The data necessary to reproduce the analyses presented here are publicly accessible and will be made available from the first author upon reasonable request. The analytic code necessary to reproduce the analyses presented in this paper is publicly accessible, available from the first author. The materials necessary to attempt to replicate the findings presented here are publicly accessible and will be made available by contacting the first author. The analyses presented here were not preregistered.
