# Supplementary material for: Cutting It Too Fine? The Factor Structure of Fine Motor Skills From Ages 5 to 10 Years
Source: Child Dev. 2025 Jul 29;96(6):1989–2005. doi: 10.1111/cdev.70016 (PMC12598455; doi:10.1111/cdev.70016)
Supplement: Supplementary file 1 — Table S1. Correlations for the younger sample at time 1 (kindergarten). Table S2. Correlations for the younger sample at time 2 (grade 1). Table S3. Correlations for the older sample at time 1 (grade 2). Table S4. Correlations for older sample at time 2 (grade 3). [file CDEV-96-1989-s001.docx]

**Table S1**

*Correlations for the Younger Sample at Time 1 (Kindergarten)*

|  | Measure | 1 | 2 | 3 | 4 | 5 | 6 | 7 | 8 | 9 |
| --- | --- | --- | --- | --- | --- | --- | --- | --- | --- | --- |
| 1 | Coin posting | - | .502^**^ | .364^**^ | .597^**^ | -.198^**^ | -.271^**^ | -.284^**^ | -.040 | -.122 |
| 2 | Bead threading |  | - | .424^**^ | .496^**^ | -.296^**^ | -.314^**^ | -.342^**^ | -.071 | -.022 |
| 3 | Weaving |  |  | - | .464^**^ | -.313^**^ | -.357^**^ | -.363^**^ | -.045 | -.044 |
| 4 | Pegboard |  |  |  | - | -.318^**^ | -.347^**^ | -.380^**^ | -.095 | -.201^**^ |
| 5 | Beery tracing |  |  |  |  | - | .465^**^ | .467^**^ | .042 | .142^*^ |
| 6 | Beery forms |  |  |  |  |  | - | .544^**^ | -.001 | .104 |
| 7 | Greek letters |  |  |  |  |  |  | - | .105 | .145^*^ |
| 8 | Rabbit speed |  |  |  |  |  |  |  | - | .372^**^ |
| 9 | Fire task |  |  |  |  |  |  |  |  | - |

*** =** *p* < .05

**** =** *p* < .01

**Table S2**

*Correlations for the Younger Sample at Time 2 (Grade 1)*

|  | Measure | 1 | 2 | 3 | 4 | 5 | 6 | 7 | 8 | 9 |
| --- | --- | --- | --- | --- | --- | --- | --- | --- | --- | --- |
| 1 | Coin posting | - | .520^**^ | .436^**^ | .619^**^ | -.397^**^ | -.203^**^ | -.244^**^ | -.173^*^ | -.266^**^ |
| 2 | Bead threading |  | - | .499^**^ | .523^**^ | -.273^**^ | -.169^*^ | -.342^**^ | -.059 | -.215^**^ |
| 3 | Weaving |  |  | - | .443^**^ | -.298^**^ | -.209^**^ | -.355^**^ | .009 | -.116 |
| 4 | Pegboard |  |  |  | - | -.352^**^ | -.178^**^ | -.254^**^ | -.225^**^ | -.249^**^ |
| 5 | Beery tracing |  |  |  |  | - | .463^**^ | .375^**^ | -.077 | .071 |
| 6 | Beery forms |  |  |  |  |  | - | .372^**^ | .019 | -.044 |
| 7 | Greek letters |  |  |  |  |  |  | - | .020 | .024 |
| 8 | Rabbit speed |  |  |  |  |  |  |  | - | .391^**^ |
| 9 | Fire task |  |  |  |  |  |  |  |  | - |

*** =** *p* < .05

**** =** *p* < .01

**Table S3**

*Correlations for the Older Sample at Time 1 (Grade 2)*

|  | Measure | 1 | 2 | 3 | 4 | 5 | 6 | 7 | 8 | 9 |
| --- | --- | --- | --- | --- | --- | --- | --- | --- | --- | --- |
| 1 | Coin posting | - | .502^**^ | .372^**^ | .603^**^ | -.293^**^ | -.232^**^ | -.407^**^ | -.185^**^ | -.357^**^ |
| 2 | Bead threading |  | - | .469^**^ | .559^**^ | -.322^**^ | -.287^**^ | -.288^**^ | -.146^**^ | -.211^**^ |
| 3 | Weaving |  |  | - | .409^**^ | -.414^**^ | -.322^**^ | -.249^**^ | -.047 | -.234^**^ |
| 4 | Pegboard |  |  |  | - | -.341^**^ | -.225^**^ | -.344^**^ | -.184^**^ | -.252^**^ |
| 5 | Beery tracing |  |  |  |  | - | .344^**^ | .203^**^ | .058 | .173^**^ |
| 6 | Beery forms |  |  |  |  |  | - | .025 | .147^*^ | .229^**^ |
| 7 | Nonword writing |  |  |  |  |  |  | - | .180^**^ | .205^**^ |
| 8 | Rabbit task |  |  |  |  |  |  |  | - | .545^**^ |
| 9 | Fire task |  |  |  |  |  |  |  |  | - |

*** =** *p* < .05

**** =** *p* < .01

**Table S4**

*Correlations for Older Sample at Time 2 (Grade 3)*

|  | Measure | 1 | 2 | 3 | 4 | 5 | 6 | 7 | 8 | 9 |
| --- | --- | --- | --- | --- | --- | --- | --- | --- | --- | --- |
| 1 | Coin posting | - | .564^**^ | .411^**^ | .682^**^ | -.191^**^ | -.219^**^ | -.162^**^ | -.236^**^ | -.356^**^ |
| 2 | Bead threading |  | - | .488^**^ | .604^**^ | -.213^**^ | -.218^**^ | -.169^**^ | -.197^**^ | -.213^**^ |
| 3 | Weaving |  |  | - | .406^**^ | -.185^**^ | -.231^**^ | -.078 | -.141^*^ | -.175^**^ |
| 4 | Pegboard |  |  |  | - | -.212^**^ | -.205^**^ | -.162^**^ | -.233^**^ | -.349^**^ |
| 5 | Beery tracing |  |  |  |  | - | .322^**^ | -.062 | .126^*^ | .104 |
| 6 | Beery forms |  |  |  |  |  | - | -.076 | .026 | .049 |
| 7 | Nonword writing |  |  |  |  |  |  | - | .120^*^ | .206^**^ |
| 8 | Rabbit task |  |  |  |  |  |  |  | - | .586^**^ |
| 9 | Fire task |  |  |  |  |  |  |  |  | - |

*** =** *p* < .05

**** =** *p* < .01
