# Supplementary material for: Cutting It Too Fine? The Factor Structure of Fine Motor Skills From Ages 5 to 10 Years
Source: Child Dev. 2025 Jul 29;96(6):1989–2005. doi: 10.1111/cdev.70016 (PMC12598455; doi:10.1111/cdev.70016)
Supplement: Supplementary file 2 — Figure S1. Screen plot for the explorative factor analyses for the kindergarten (above) and grade 2 (below) randomly split samples. [file CDEV-96-1989-s002.docx]

*Figure S1. Scree plot for the explorative factor analyses for the kindergarten (above) and grade 2 (below) randomly split samples.*
