# Supplementary material for: Cutting It Too Fine? The Factor Structure of Fine Motor Skills From Ages 5 to 10 Years
Source: Child Dev. 2025 Jul 29;96(6):1989–2005. doi: 10.1111/cdev.70016 (PMC12598455; doi:10.1111/cdev.70016)
Supplement: Supplementary file 3 — Supinfo [file CDEV-96-1989-s003.docx]

**Table: Greek Letters Used in Copying Task in the Younger Cohort**

| α | | α | |
| --- | --- | --- | --- |
|  | |  | |
| α | | β | |
|  | |  | |
| χ | | δ | |
|  |  | | |
| ε | φ | | |
|  |  | | |
| γ | η | | |
|  |  | | |
| ι | ϕ | | |
|  |  | | |
| κ | | λ | |
|  | |  | |
| μ | | ν | |
|  | |  | |
| o | | π | |
|  | |  | |
|  | |  | |
| θ | ρ | | |
|  |  | | |
| σ | τ | | |
|  |  | | |
| υ | ϖ | | |
|  |  | | |
| ω | | | ξ |
|  | | |  |
| ψ | | | ζ |
|  | | |  |

**Table: Nonword Copying Task Stimuli Used in the Older Cohort**

| foo | Blost |
| --- | --- |
|  |  |
| Hoot | Schwusch |
|  |  |
| zier | But |
|  |  |
| Trün | gert |
|  |  |
| Wieb | jot |
|  |  |
| Hentist | metter |
|  |  |
| soktor | Onarch |
|  |  |
| Gunger | Brucker |
|  |  |
| Milber | Lallett |
|  |  |
| Balopp | Mofen |
|  |  |
| Pakobel | therigiös |
|  |  |
| Inlio | Barano |
|  |  |
| Kosiminz | renite |
|  |  |
| quaduktrisch | Miktanie |
|  |  |
| plauferfant | Disaform |
|  |  |
